# Supplementary figures and images for: Interrelation Between Pathoadaptability Factors and Crispr-Element Patterns in the Genomes of Escherichia coli Isolates Collected from Healthy Puerperant Women in Ural Region, Russia
Source: Pathogens. 2024 Nov 14;13(11):997. doi: 10.3390/pathogens13110997 (PMC11597047; doi:10.3390/pathogens13110997)

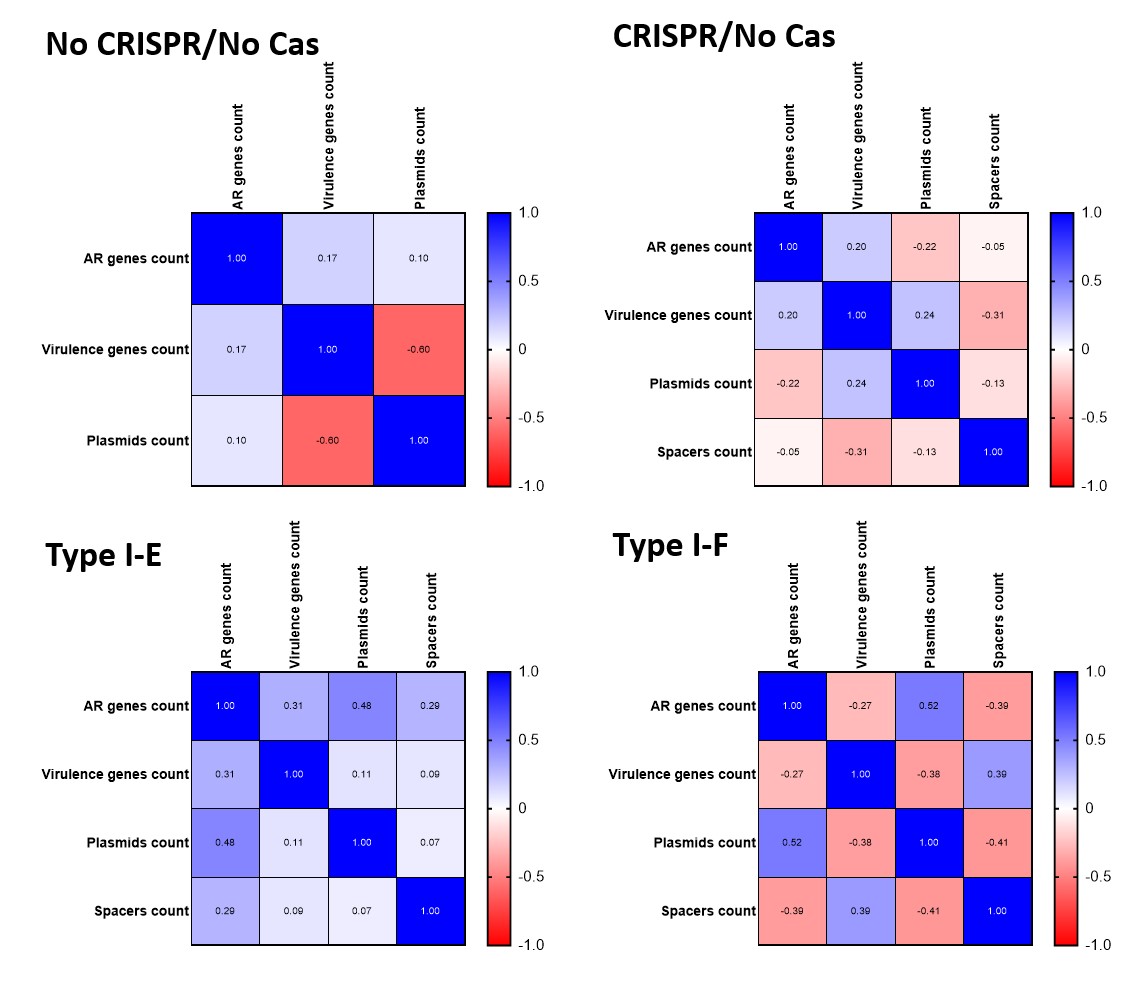

Supplement: Supplementary file 1 [file pathogens-13-00997-s001.zip › Figure S1.jpg]

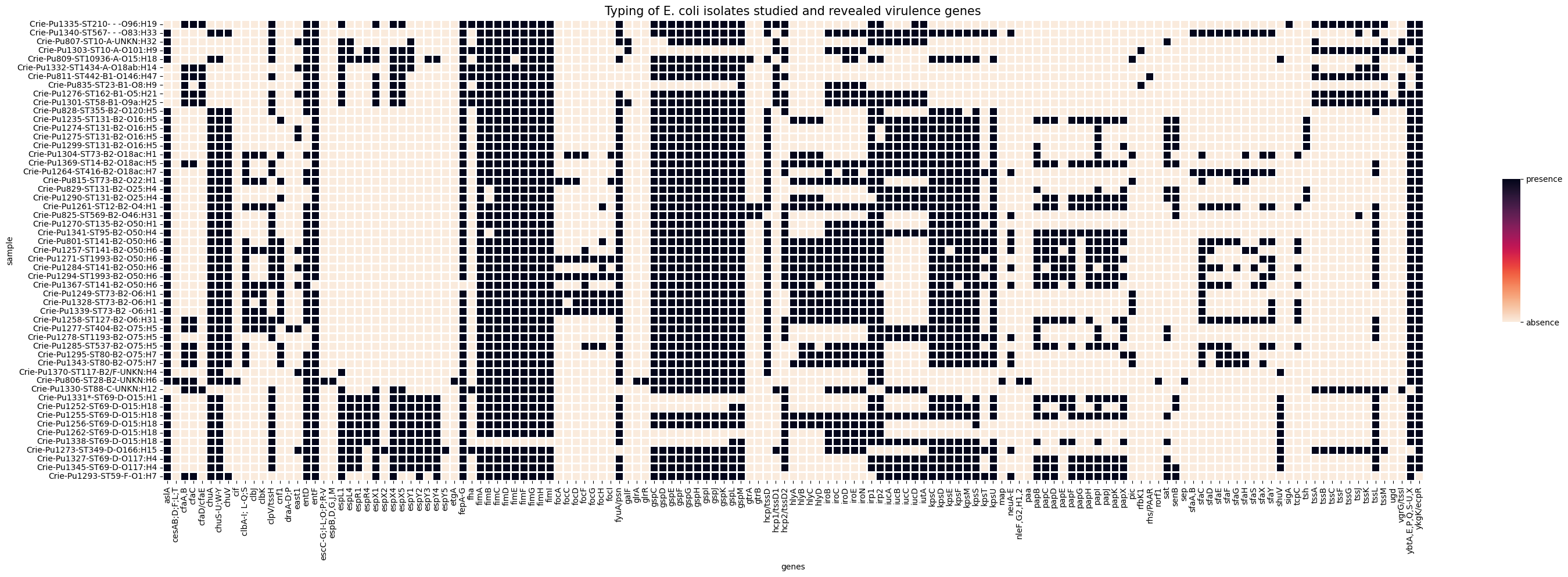

Supplement: Supplementary file 1 [file pathogens-13-00997-s001.zip › Table_S2.png]

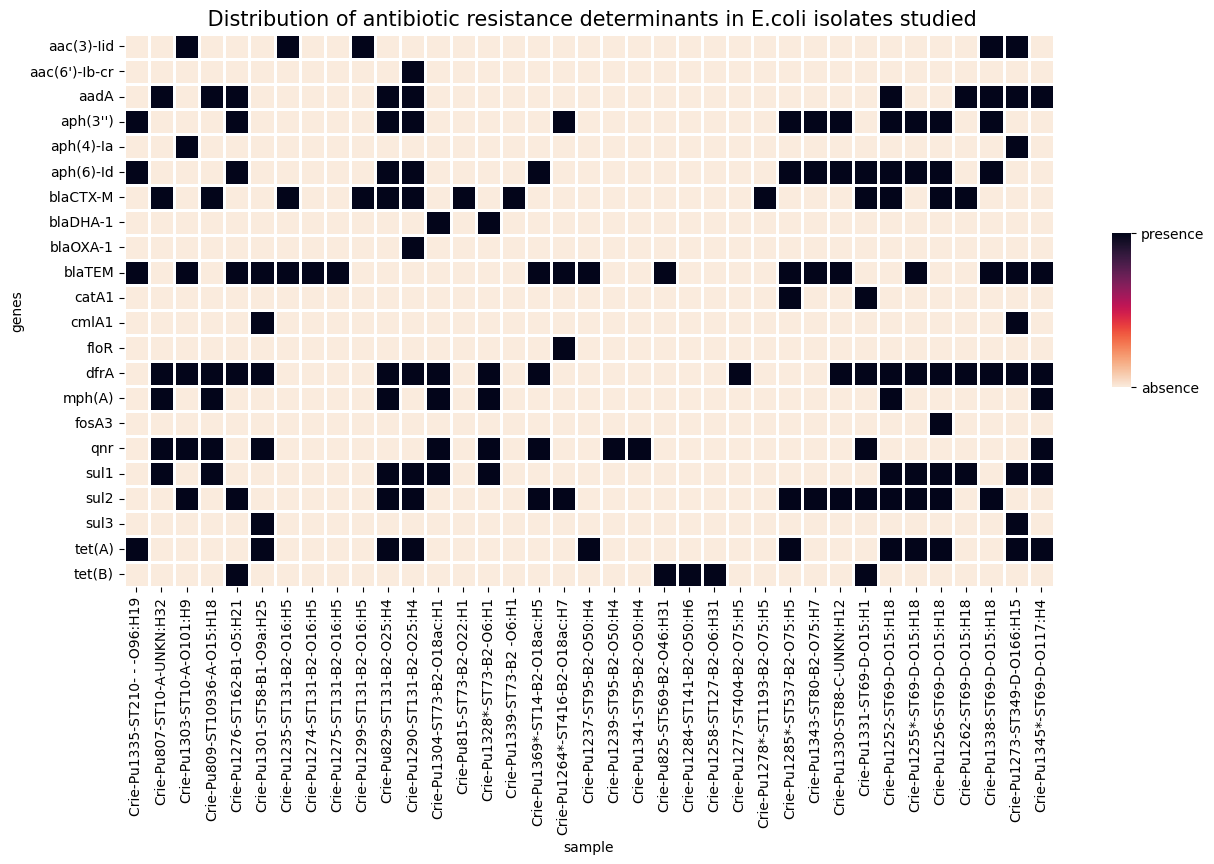

Supplement: Supplementary file 1 [file pathogens-13-00997-s001.zip › Table_S4.png]

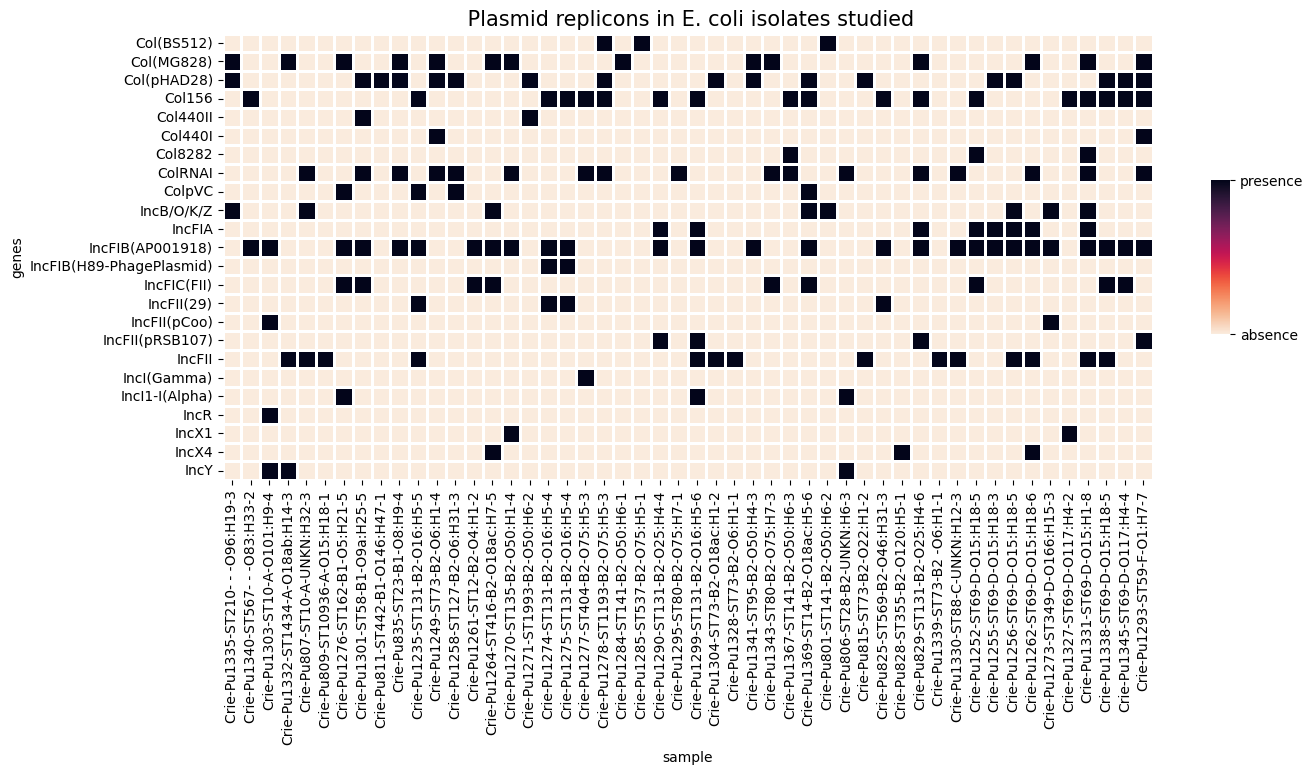

Supplement: Supplementary file 1 [file pathogens-13-00997-s001.zip › Table_S5.png]
